# Menthol as an Ergogenic Aid for the Tokyo 2021 Olympic Games: An Expert-Led Consensus Statement Using the Modified Delphi Method

**DOI:** 10.1007/s40279-020-01313-9

**Published:** 2020-07-04

**Authors:** M. J. Barwood, O. R. Gibson, D. J. Gillis, O. Jeffries, N. B. Morris, J. Pearce, M. L. Ross, C. Stevens, K. Rinaldi, S. N. Kounalakis, F. Riera, T. Mündel, M. Waldron, R. Best

**Affiliations:** 1 Department of Sport, Health and Nutrition, Leeds Trinity University, Brownberrie Lane, Horsforth, Leeds, LS18 5HD UK; 2 Centre for Human Performance, Exercise and Rehabilitation (CHPER), Department Life Sciences, Division of Sport, Health and Exercise Sciences, Brunel University London, Kingston Lane, Uxbridge, UB8 3PH UK; 3 Human Performance Laboratory, Department of Sport and Movement Science, Salem State University, Salem, MA 01970 USA; 4 School of Biomedical, Nutritional and Sport Sciences, Faculty of Medical Sciences, Newcastle University, Catherine Cookson Building, Newcastle Upon Tyne, NE2 4HH UK; 5 Department of Nutrition, Exercise and Sports, University of Copenhagen, 2100 Copenhagen, Denmark; 6 Performance Nutrition Technical Lead, High Performance Sport New Zealand, Auckland, New Zealand; 7 Australian Institute of Sport, Bruce, 2617 Australia; 8 Mary Mackillop Institute for Health Research, Australian Catholic University, Melbourne, 3000 Australia; 9 School of Health and Human Sciences, Southern Cross University, Hogbin Dr, Coffs Harbour, NSW 2450 Australia; 10 Laboratoire ACTES (EA3596), Université des Antilles et de la Guyane, BP 250, 97157 Pointe-à-Pitre, France; 11 Arkea Samsic Pro Cycling Team, 35170 Bruz, France; 12 Faculty of Physical and Cultural Education, Evelpidon Hellenic Army Academy, Vari, Greece; 13 UPRES EA 35-96, UFR-STAPS, Université des Antilles et de la Guyane, BP 250, 97157 Pointe à Pitre Cedex, France; 14 Laboratoire Performance Santé Altitude, Université de Perpignan Via Domitia, UFR Sciences et Techniques des Activités Physiques et Sportives, 7 avenue Pierre de Coubertin, 66120 Font-Romeu, France; 15 School of Sport Exercise and Nutrition, Massey University, Palmerston, New Zealand; 16 College of Engineering, Applied Sports Science Technology and Medicine Research Centre (A-STEM), Swansea University Bay Campus, Swansea, Wales UK; 17 School of Science and Technology, University of New England, Armidale, NSW Australia; 18 Centre for Sport Science and Human Performance, Waikato Institute of Technology, Hamilton, 3200 New Zealand; 19 School of Health and Social Care, Teesside University, Middlesbrough, Tees Valley, TS1 3BX UK

## Abstract

**Introduction:**

Menthol topical application and mouth rinsing are ergogenic in hot environments, improving performance and perception, with differing effects on body temperature regulation. Consequently, athletes and federations are beginning to explore the possible benefits to elite sport performance for the Tokyo 2021 Olympics, which will take place in hot (~ 31 °C), humid (70% RH) conditions. There is no clear consensus on safe and effective menthol use for athletes, practitioners, or researchers. The present study addressed this shortfall by producing expert-led consensus recommendations.

**Method:**

Fourteen contributors were recruited following ethical approval. A three-step modified Delphi method was used for voting on 96 statements generated following literature consultation; 192 statements total (96/96 topical application/mouth rinsing). Round 1 contributors voted to “agree” or “disagree” with statements; 80% agreement was required to accept statements. In round 2, contributors voted to “support” or “change” their round 1 unaccepted statements, with knowledge of the extant voting from round 1. Round 3 contributors met to discuss voting against key remaining statements.

**Results:**

Forty-seven statements reached consensus in round 1 (30/17 topical application/rinsing); 14 proved redundant. Six statements reached consensus in round 2 (2/4 topical application/rinsing); 116 statements proved redundant. Nine further statements were agreed in round 3 (6/3 topical application/rinsing) with caveats.

**Discussion:**

Consensus was reached on 62 statements in total (38/24 topical application/rinsing), enabling the development of guidance on safe menthol administration, with a view to enhancing performance and perception in the heat without impairing body temperature regulation.

## Key Points

**Table:** 

Menthol topical application and mouth rinsing are ergogenic in hot environments, improving performance and perception, with differing effects on body temperature regulation.
Consequently, athletes and federations are beginning to explore the possible benefits to elite sport performance for the Tokyo 2021 Olympics, which will take place in hot (~ 31 °C), humid (70% RH) conditions.
Menthol topical application and mouth rinsing are ergogenic in endurance activities and show promise as an intervention to enhance other sport performance.
This consensus statement provides guidance on the safe and effective use of menthol for athletes, practitioners, and researchers.

## Introduction

Menthol is a naturally occurring cyclic terpene alcohol that is extracted from plants of the *Mentha* genus, e.g., peppermint and corn mint [25]. Presenting in eight forms, the (-)isomer is responsible for menthol’s characteristically fresh aroma, taste, and cooling sensation when applied to mucous membranes or the skin, with its effects inversely proportional to the thickness of the membrane to which it is applied [66, 72]. Menthol elicits these sensations by primarily stimulating the membrane bound ion channel transient receptor potential melastatin 8 (TRPM-8), mirroring temperature change within the range of 8–28 °C [58]. Stimulation of these receptors during periods of heat stress has consistently been shown to improve thermal comfort and decrease thermal sensation [38, 66]. Further downstream effects of menthol topical application or mouth rinsing may include improvements in subjective nasal patency [21, 22], alterations in blood flow [16, 39], altered body temperature regulation [44], and attenuation of thirst [22]. Given its unique ability to evoke an array of physiological and perceptual responses, menthol is widely used in commercial products, ranging from topical analgesics to oral hygiene products. More recently, scientists have begun to implement menthol focussed interventions in sport and exercise settings. Increasing attention has been focussed on how these strategies can be employed safely and effectively in hot environments that relate to the demands of forthcoming global sporting events, such as the Olympic Games in 2021 to be held in Tokyo, the recent IAAF World Athletic Championships in 2019 in Qatar and forthcoming World Cup football in 2022 also in Qatar, where thermal challenges will impact athletic performances. Accordingly, a body of research is accumulating on a wide range of menthol focussed interventions.

Menthol can be applied topically via creams, gels, or sprays [6, 8, 13, 29, 31, 32, 44], with timing of administration altered to suit the aim of the exercise bout and nature of menthol topical application. For instance, menthol applied as a spray prior to or repeatedly throughout an exercise bout has been shown to improve thermal comfort and sensation, but may alter athletes’ sweat rate [8, 29, 30, 44], potentially presenting a conflict in thermoregulatory drives. Oral application (e.g., mouth rinsing) and ingestion of menthol, on the other hand, stimulate the mandibular and maxillary branches of the trigeminal nerve, which are predominantly responsible for detection of temperature and nociceptive stimuli across the face and within the oral cavity [41, 43], thereby imparting a localised cooling or analgesic effect. Menthol topical application to the skin and menthol mouth rinsing have been predominantly investigated as the mode of administration during exercise [24, 28, 37, 52, 69], although a handful of papers have also assessed co-ingestion or the addition of menthol to beverages of varying temperatures [60, 61, 69].

An increase in menthol use by athletes is expected over the coming decade, with many major sporting events taking place at venues that pose significant heat challenges. Ameliorating athletes’ perception of heat stress by improving thermal comfort or attenuating elevations in thermal sensation will likely be of benefit to performance, could alter pacing, but may increase the risk of heat illness in some athletes. The potential risks of oral or topical menthol application in the heat are not yet fully understood. The safety and possible toxicity of menthol containing products must also be considered, independent of mode of application, with factors such as concentration [11], surface area of topical application [13, 31, 44, 72], and sources of possible contamination [20] all pertinent considerations for athletes, practitioners, and support staff. Despite the accumulating body of evidence describing the experimental effects of menthol, there is little agreement on how best to safely administer it as an ergogenic aid.

Accordingly, the current project aimed to generate an expert consensus statement on: (1) the evidence base underpinning the ergogenic effects of single and repeated use of menthol topical application and mouth rinsing in activity types (sports) that are typical to the 2021 Tokyo Olympic Games; (2) to characterise the population(s) in which this evidence base has been established; (3) to describe the reliable psychophysiological effects of topical application and mouth rinsing based on published data; (4) to describe the possible health-related consequences of menthol use in temperate and hot environments; (5) to consider if menthol topical application and mouth rinsing are within the “spirit and ethos” of Olympic sport; (6) to review the quality of published evidence underpinning the above observations. We subsequently provide recommendations for menthol use by athletes, practitioners, and researchers.

## Method

Prior ethical approval for the study was granted by the Waikato Institute of Technology Human Ethics Research Group. The consensus process utilised a three-step modified Delphi method [18, 19, 23], which took place between April and July 2019. The consensus was structured using domain 1 of the Appraisal of Guidelines Research and Evaluation (AGREE) reporting checklist [14], providing the structure for the “Scope and Purpose” of the consensus by considering the objectives, questions, and the target population for the menthol-based interventions. A modified Delphi method was used to conduct the data collection as it is recognised as a reliable method of reaching a consensus for a defined research problem [e.g. 49, 53]. The method employs three iterative rounds of voting, with systematic progression between rounds to reach a final consensus. Initially, a series of statements were developed focussing on the use of menthol topical application and mouth rinsing in the sporting setting in relation to sport performance, perception, and thermoregulation. These statements were organised into sub-categories relating to (1) activity type, (2) population; (3) experimental effects; (4) health effects; (5) spirit of the sport; and (6) levels of evidence. The first two rounds of voting were undertaken independently by each expert, whilst following standardised instructions, and were coordinated by email. Similar to Eubank et al. [23], the present study modified the final round to include a face-to-face meeting of experts to undertake consensus voting. This allowed contributors to discuss their justifications, provide clarification, and offer any required caveats to support or reject any remaining statements.

### Panel Selection

Between five and ten contributors are considered adequate to formulate a consensus group [48]. Inclusion criteria were: (1) one or more lead author publication(s) examining menthol topical application or mouth rinsing as an intervention to influence sport performance, thermoregulation or perception in a hot environment; (2) a PhD/research programme focussed on menthol use in sport or (3) recognition as a research leader or informed practitioner in the above area. Panel members primarily represented exercise scientists, applied scientists, and practitioners, and were drawn from the international scientific community. Once panel members were contacted, the goals and processes of the study were explained using standardised terms and written, informed consent was gained by return email. It should be noted that the assembled panel is not an exhaustive list of all persons who have explored menthol as an ergogenic aid. Rather, it represents a panel of informed experts who meet one or more of the above criteria.

### Systematic Reviews of Literature

Previous modified Delphi studies have typically included systematic literature searches to generate the consensus statements that form the basis for voting [23]. Given that two of the most recent systematic literature searches had been conducted by members of the lead authorship team [38, 66], a further literature search was not necessary. The consensus statements were formed on the basis of these experiences and observations gained from administering menthol in the laboratory and field setting. Accordingly, 96 statements were generated in relation to the two application modalities (i.e., menthol topical application and menthol mouth rinsing). Within each modality, the statements were considered against the extant published evidence underpinning single (i.e., 48 statements within each modality) and repeated (i.e., 48 statements within each modality) effects of menthol topical application and mouth rinsing. Therefore, a total of 192 statements were entered for voting into round 1.

### Round 1

The draft document containing the list of statements was circulated to the 14 panel members, accompanied by a clear timeline, method, statement of the research aims, and expected project outcomes. Each contributor was asked to vote by marking “agree” or “disagree” beside each statement. If they neither agreed nor disagreed, they were instructed to leave the space blank. Statements required 80% agreement, which was reached when (a) 11 or more of the entire consensus group voted or (b) when 10 or more votes were cast against a statement and the 80% threshold was reached. These cut-off criteria were decided in accordance with Lynn [48], who suggested that 80% agreement was required for an item to achieve content validity. Statements receiving more than ten blank responses were removed following round 1, assuming redundancy (i.e., insufficient evidence for voting). Following statement agreement and removal of redundant statements, any remaining statements were forwarded to round 2. A 24 day turnaround was applied to round 1 voting.

### Round 2

The list of statements that received at least one vote, but did not reach consensus from round 1, following anonymisation and randomisation by a researcher independent to the expert panel, was emailed to all contributors from round 1. The same voting method was used as for round 1 but with the knowledge of the group votes from round 1 reproduced against each remaining statement. Contributors were encouraged to consider their initial response to a given statement, review the balance of voting from round 1, and then either “support” or “change” the round 1 response. If they changed their response in light of wider group responses, they were encouraged to report the conditional reason describing why the change was permissible. A 19-day turn around was applied to round 2 voting. Final responses were analysed as described in round 1.

### Round 3

Round 3 comprised a face-to-face meeting as a supplementary activity at the 2019 International Conference on Environmental Ergonomics, Amsterdam, The Netherlands. Those members of the expert panel who were not able to attend the meeting were invited to attend by video call. Round 3 voting did not preserve anonymity and contributors were encouraged to discuss only the remaining statements that had a minimum of ten votes cast against them, but did not reach the 80% threshold. Decisions were made to retain, modify (i.e., add a caveat) or eliminate a statement. An independent chair was recruited and appointed to oversee the round 3 discussions.

## Results

### Panel Participation

Fourteen panel members contributed to round 1. Twelve contributors produced returns for round 2 of voting. Ten contributors, five in person and five via video call, attended round 3 of voting. Hence, according to the criteria, we had established* a priori* [48], and the panel was quorate and could reach a consensus on a given statement throughout all rounds of voting.

### Round 1

**Fig. 1:**
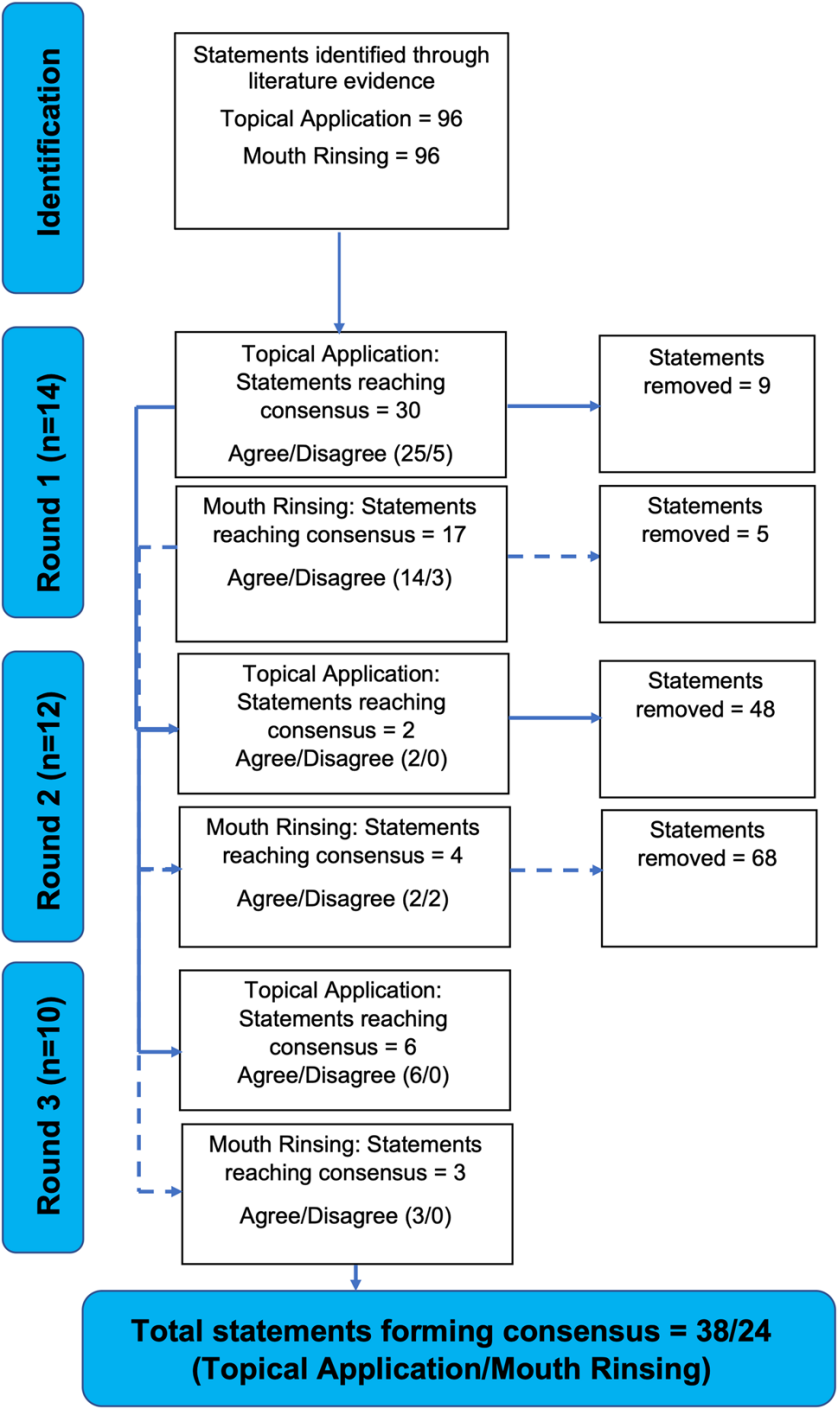
Progression in consensus voting describing the accepted and removed statements between rounds 1 and 3. Solid lines indicate the advancement and removal of statements between rounds for menthol topical application and dashed lines indicate the advancement and removal of statements between rounds for menthol mouth rinsing

Figure 1 summarises the progression of the consensus statement across rounds 1–3. After round 1 of voting was complete, a total of 30 statements (25 agree, 5 disagree) reached a consensus in relation to menthol topical application. A total of 17 statements (14 agree, 3 disagree) reached a consensus in relation to menthol mouth rinsing. A total of 14 statements (9 topical application, 5 mouth rinsing) were considered redundant on grounds of lack of evidence to interrogate them (i.e., no votes were cast against them).

### Round 2

A total of 57 statements were entered into round 2 for menthol topical application and 75 statements were entered for menthol mouth rinsing. After round 2 voting was complete, a further 2 statements (2 agree, 0 disagree) reached a consensus in relation to menthol topical application. A total of 4 statements (2 agree, 2 disagree) reached a consensus in relation to mouth rinsing. A total of 116 statements (48 topical application, 68 mouth rinsing) were considered redundant on grounds of lack of evidence to interrogate them based on the feedback comments in round 2.

### Round 3

A total of 6 statements were entered into round 3 for menthol topical application and 3 statements were entered for menthol mouth rinsing. All of the statements reached consensus following revision or the addition of a caveat. An itemised list of the statements that reached consensus are included in Tables 1, 2, 3, 4, 5, 6, 7, 8 and 9 in relation to each of the six domains that were examined.

**Table 1:** Single and repeated topical menthol application—accepted consensus statements (a–g) from round 1, 2, and 3 for (1) activity type and (2) population

Item	Question no.	Domain	Context	Statement	Agree/disagree	Round achieved	Caveat
a	1	(1) Activity type	Endurance (e.g., athletic exercise efforts lasting > 2.5 min)	Single topical Menthol application enhances the performance of this activity	Agree	3	At high concentration (e.g., 8% concentration; e.g., Schlader et al. [62]) if test is considered valid representation of athletic event
b	11	(1) Activity type	Endurance (e.g., athletic exercise efforts lasting > 2.5 min)	Repeated topical Menthol application enhances the performance of this activity	Agree	1	Barwood et al. [8] saw performance effect at fixed power output to exhaustion; if test is considered valid representation of athletic event
c	22	(2) Population	Recreationally active	Single topical Menthol application enhances the performance of this population	Agree	1	–
d	23	(2) Population	Trained	Single topical Menthol application enhances the performance of this population	Agree	1	–
e	26	(2) Population	Males	Single topical Menthol Application enhances the performance of this population	Agree	1	–
f	31	(2) Population	Trained	Repeated topical Menthol application enhances the performance of this population	Agree	1	–
g	34	(2) Population	Males	Repeated topical Menthol application enhances the performance of this population	Agree	1	–

**Table 2:** Single and repeated topical menthol application—accepted consensus statements (a–j) from round 1, 2, and 3 for (3) Experimental Effects

Item	Question no.	Domain	Context	Statement	Agree/disagree	Round achieved	Caveat
a	37	(3) Experimental effects	Improves thermal discomfort (i.e., participants feel more comfortable)	Single topical Menthol Application reliably induces the following experimental effects during exercise in the heat	Agree	1	–
b	38	(3) Experimental effects	Lowers thermal sensation (i.e., participants feel cooler on topical application)	Single topical Menthol Application reliably induces the following experimental effects during exercise in the heat	Agree	1	-
c	39	(3) Experimental effects	Lowers rating of perceived exertion for a given workload	Single topical Menthol Application reliably induces the following experimental effects during exercise in the heat	Agree	1	–
d	40	(3) Experimental effects	Alters behavioural thermoregulation (i.e., participants can exercise for longer/work at a higher intensity)	Single topical Menthol Application reliably induces the following experimental effects during exercise in the heat	Agree	3	In fixed intensity/perceptual or tolerance based protocols
e	41	(3) Experimental effects	Alters reflex thermoregulatory heat defense responses of sweating	Single topical Menthol Application reliably induces the following experimental effects during exercise in the heat	Agree	3	At 0.20% menthol spray descriptively decreasedsweat rate in Gillis et al. [29] and 0.80% menthol in Lee et al. [47] delayed sweat onset. Kounalakis et al. [44] showed delayed sweating in swimmers/non swimmers. Surface area and concentration-dependent.
f	42	(3) Experimental effects	Alters reflex thermoregulatory heat defense responses of peripheral blood flow	Single topical Menthol Application reliably induces the following experimental effects during exercise in the heat	Agree	1	–
g	49	(3) Experimental effects	Is body surface area dependent for the magnitude of its experimental effects	Single topical Menthol Application reliably induces the following experimental effects during exercise in the heat	Agree	1	–
h	50	(3) Experimental effects	Is concentration-dependent for the magnitude of its experimental effects	Single topical Menthol Application reliably induces the following experimental effects during exercise in the heat	Agree	1	–
i	52	(3) Experimental effects	Lowers thermal sensation (i.e., participants feel cooler on topical application)	Repeated topical Menthol Application reliably induces the following experimental effects during exercise in the heat	Agree	1	–
j	54	(3) Experimental effects	Alters behavioural thermoregulation (i.e., participants can exercise for longer/work at a higher intensity)	Repeated topical Menthol Application reliably induces the following experimental effects during exercise in the heat	Agree	1	–

**Table 3:** Single and repeated topical menthol application—accepted (a–h) from round 1, 2, and 3 for (4) Health Effects

Item	Question no.	Domain	Context	Statement	Agree/disagree	Round achieved	Caveat
a	65	(4) Health effects	Is safe at low concentrations	Single topical Menthol application	Agree	1	–
b	66	(4) Health effects	Is harmful at high concentrations	Single topical Menthol application	Agree	1	–
c	68	(4) Health effects	Requires specialist training for safe administration	Single topical Menthol application	Agree	3	When the product is not produced by a food or medically screened national or international standard (i.e., in a lab from "Raw" ingredients). If products not used as instructed by manufacturer. Use of food-grade menthol. Caution required (e.g. refer to specialist when not using a commercially available product)
d	69	(4) Health effects	Use is widespread in sporting activities	Single topical Menthol application	Agree	1	–
e	70	(4) Health effects	Is safe at low concentrations	Repeated topical Menthol application	Agree	1	–
f	71	(4) Health effects	Is harmful at high concentrations	Repeated topical Menthol application	Agree	2	
g	73	(4) Health effects	Requires specialist training for safe administration	Repeated topical Menthol Application	Agree	3	When the product is not produced by a food or medically screened national or international standard (i.e., in a lab from "Raw" ingredients). If products not used as instructed by manufacturer. Use of food-grade menthol. Caution required (e.g., refer to specialist when not using a commercially available product)
h	74	(4) Health effects	Use is widespread in sporting activities	Repeated topical Menthol Application	Disagree	1

**Table 4:** Single and repeated topical menthol application—accepted consensus statements (a–h) from round 1, 2, and 3 for (5) Spirit of the Sport

Item	Question no.	Domain	Context	Statement	Agree/disagree	Round achieved	Caveat
a	75	(5) Spirit of the sport	Has the potential to enhance or is known to enhance sport performance	Single topical Menthol application	Agree	1	–
b	76	(5) Spirit of the sport	It represents an actual or potential health risk to the participant	Single topical Menthol application	Agree	3	Yes, we referred to U.K. toxic substance database report and published cases. Add delimitations around likely concentration for use in sport
c	77	(5) Spirit of the sport	It violates the spirit of the sport	Single topical Menthol application	Disagree	1	
d	78	(5) Spirit of the sport	Gives an unfair advantage	Single topical Menthol application	Disagree	1	
e	79	(5) Spirit of the sport	Has the potential to enhance or is known to enhance sport performance	Repeated topical Menthol Application	Agree	1	–
f	80	(5) Spirit of the sport	It represents an actual or potential health risk to the participant	Repeated topical Menthol application	Agree	2	
g	81	(5) Spirit of the sport	It violates the spirit of the sport	Repeated topical Menthol application	Disagree	1	
h	82	(5) Spirit of the sport	Gives an unfair advantage	Repeated topical Menthol application	Disagree	1

**Table 5:** Single and repeated topical menthol application—accepted (a–e) from round 1, 2, and 3 for (6) Levels of Evidence

Item	Question no.	Domain	Context	Statement	Agree/disagree	Round achieved	Caveat
a	83	(6) Levels of Evidence	Laboratory settings	The effects of single topical Menthol Application have most been established in:	Agree	1	–
b	85	(6) Levels of Evidence	With the presence of adequate controls	The effects of single topical Menthol Application have most been established in:	Agree	1	–
c	87	(6) Levels of Evidence	In within Subject Designs	The effects of single topical Menthol Application have most been established in:	Agree	1	–
d	90	(6) Levels of Evidence	Laboratory settings	The effects of repeated topical Menthol Application have most been established in:	Agree	1	–
e	94	(6) Levels of Evidence	In within Subject Designs	The effects of repeated topical Menthol Application have most been established in:	Agree	1	–

**Table 6:** Single and repeated menthol mouth rinsing—accepted consensus statements (a–d) from round 1, 2, and 3 for (1) activity type and (2) population

Item	Question no.	Domain	Context	Statement	Agree/disagree	Round achieved	Caveat
a	11	(1) Activity type	Endurance (e.g., athletic exercise efforts lasting > 2.5 min)	Repeated occasions of Menthol rinsing enhance the performance of this activity	Agree	1	–
b	30	(2) Population	Recreationally active	Repeated occasions of Menthol rinsing enhance the performance of this population	Agree	1	–
c	31	(2) Population	Trained	Repeated occasions of Menthol rinsing enhance the performance of this population	Agree	1	–
d	34	(2) Population	Males	Repeated occasions of Menthol rinsing enhance the performance of this population	Agree	1	–

**Table 7:** Single and repeated menthol mouth rinsing—accepted consensus statements (a–h) from round 1, 2, and 3 for (3) experimental effects

Item	Question no.	Domain	Context	Statement	Agree/disagree	Round achieved	Caveat
a	38	(3) Experimental effects	Lowers thermal sensation (i.e. participants feel cooler on topical application)	Single occasions of Menthol Rinsing reliably induces the following experimental effects during exercise in the heat	Agree	1	–
b	51	(3) Experimental effects	Improves thermal discomfort (i.e., participants feel more comfortable)	Repeated occasions of Menthol Rinsing reliably induces the following experimental effects during exercise in the heat	Agree	1	–
c	52	(3) Experimental effects	Lowers thermal sensation (i.e., participants feel cooler on topical application)	Repeated occasions of Menthol Rinsing reliably induces the following experimental effects during exercise in the heat	Agree	1	–
d	53	(3) Experimental effects	Lowers rating of perceived exertion for a given workload	Repeated occasions of Menthol Rinsing reliably induces the following experimental effects during exercise in the heat	Agree	1	–
e	54	(3) Experimental effects	Alters behavioural thermoregulation (i.e., participants can exercise for longer/work at a higher intensity)	Repeated occasions of Menthol Rinsing reliably induces the following experimental effects during exercise in the heat	Agree	1	–
f	57	(3) Experimental effects	Alters skin temperature	Repeated occasions of Menthol Rinsing reliably induces the following experimental effects during exercise in the heat	Disagree	1	–
g	59	(3) Experimental effects	Increases the risk of heat illness	Repeated occasions of Menthol Rinsing reliably induces the following experimental effects during exercise in the heat	Agree	3	Urge caution when using particularly in elite/highly motivated groups
h	64	(3) Experimental effects	Is concentration-dependent for the magnitude of its experimental effects	Repeated occasions of Menthol Rinsing reliably induces the following experimental effects	Agree	2	–

**Table 8:** Single and repeated menthol mouth rinsing—accepted consensus statements (a–d) from round 1, 2, and 3 for (4) health effects

Item	Question no.	Domain	Context	Statement	Agree/disagree	Round achieved	Caveat
a	69	(4) Health effects	Use is widespread in sporting activities	Single occasions of Menthol rinsing	Disagree	2	–
b	70	(4) Health effects	Is safe at low concentrations	Repeated occasions of Menthol rinsing	Agree	1	–
c	73	(4) Health effects	Requires specialist training for safe preparation and administration	Repeated occasions of Menthol rinsing	Agree	3	When the product is not produced by a food or medically screened national or international standard (i.e. in a lab from "Raw" ingredients). If products not used as instructed by manufacturer. Use of food-grade menthol. Caution required for preparing mouth rinses as no commercial product is available (e.g., refer to dietician/nutritionists/specialists)
d	74	(4) Health effects	Use is widespread in sporting activities	Repeated occasions of Menthol rinsing	Disagree	2	–

**Table 9:** Single and repeated menthol mouth rinsing—accepted consensus statements (a–h) from round 1, 2 and 3 for (5) spirit of the sport and (6) levels of evidence

Item	Question no.	Domain	Context	Statement	Agree/disagree	Round achieved	Caveat
a	79	(5) Spirit of the sport	Has the potential to enhance or is known to enhance sport performance	Repeated occasions of Menthol rinsing	Agree	1	–
b	80	(5) Spirit of the sport	It represents a potential risk to the participant	Repeated occasions of Menthol rinsing	Agree	3	Yes, we referred to U.K. toxic substance database report and published cases. Add delimitations around likely concentration for use in sport
c	81	(5) Spirit of the sport	It violates the spirit of the sport	Repeated occasions of Menthol rinsing	Disagree	1	–
d	82	(5) Spirit of the sport	Gives an unfair advantage	Repeated occasions of Menthol rinsing	Disagree	1	–
e	83	(6) Levels of evidence	Laboratory settings	The effects of single occasions of Menthol rinsing have most been established in:	Agree	1	–
f	90	(6) Levels of evidence	Laboratory settings	The effects of repeated Menthol rinsing have most been established in:	Agree	1	–
g	92	(6) Levels of evidence	With the presence of adequate controls	The effects of repeated Menthol rinsing have most been established in:	Agree	1	–
h	94	(6) Levels of evidence	In within-subject designs	The effects of repeated Menthol rinsing have most been established in:	Agree	1	–

## Discussion

We aimed to generate an expert-led consensus statement using a systematic and rigorous method on the evidence base underpinning the ergogenic effect of menthol topical application and mouth rinsing in activity types (sports) that are typical to the 2021 Tokyo Olympic Games; our findings also apply to events and environments that are similar. In doing so, we sought to characterise the population(s) in which this evidence base has been established and describe the reliable psychophysiological effects of topical application and mouth rinsing based on published data. We also describe the possible health-related consequences of menthol use in temperate and hot environments and our view on whether these interventions are within the “spirit and ethos” of the Olympic movement. Finally, we consider the quality of published evidence underpinning the above observations with a view to applying the findings to elite sport. In sections (i) to (vi), we provide recommendations for menthol use by athletes, practitioners, and researchers with the agreed consensus statements reproduced for clarity in Tables 1, 2, 3, 4, 5, 6, 7, 8, and 9.

### Activity Type

The panel agreed that endurance activity (primarily aerobic exercise lasting > 2.5 min) can be improved by single and repeated menthol topical application (Statement—Table 1a, b; [8, 63]). However, it was clear that the mode of application, whether topically or orally, and nature of the endurance activity, may determine its ergogenic potential. For example, single topical application of a menthol paste (8% concentration) to the face extends exercise performance by 17% during an RPE-clamp protocol [63]. Spray application (100 mL of 0.20% menthol spray) whilst apparently ineffective when applied once [4, 5], can increase work output at a fixed intensity time to exhaustion [TTE] test by 48% when applied repeatedly [8]. In more ecologically valid protocols such as time trials [TT], which attempt to replicate competition, again single topical applications of menthol spray [e.g., 4, 5] conferred no ergogenic effect. It should be noted that the practical and logistical difficulties that come with repeated topical application of menthol in competition may limit its use to pre-event application only.

There was consensus regarding the efficacy of orally applied menthol to improve endurance performance when used repeatedly throughout exercise (Statement—Table 6a; [24, 52, 69]). These effects are consistent across a number of exercise modalities (e.g., running [69] and cycling [52]). Enhanced performance has also been observed across a number of exercise tests such as fixed intensity exhaustive tests [52], RPE-clamp protocols [24], and TT [69] with mouth rinses typically applied at 5–10 min intervals in these studies. Alternative approaches to oral delivery of menthol have investigated co-ingestion with other beverages at varying temperatures [60, 61, 71]. Whilst the co-ingestion of menthol may produce performance effects in a temperature dependent manner [10], we cannot be certain if these effects are additive or synergistic due to the coincidental stimulation and inhibition of cold and warm thermoreceptors, respectively, by the ingested fluid [36]. Therefore, to achieve consensus on the isolated uses of menthol in sport, we have not further examined co-ingestion strategies.

The panel did not reach consensus for a range of other intermittent [28], dynamic and explosive activities, fine motor movements, or team-based competitive sports, largely due to insufficient research in the field. There remains work to be done in these areas to advance our understanding of the effectiveness of menthol.

#### Section Summary and Practical Recommendations

- Topically applied menthol can improve TTE performance for both single (high concentration) and repeated topical applications.
- Orally applied menthol improves TTE and TT performance when used repeatedly throughout the exercise bout; there is less supporting evidence regarding singular topical application.
- There is not sufficient evidence currently to support menthol’s topical application in team-based sports; this is a promising avenue for future research.

### Population

The ergogenic benefits associated with menthol have primarily been established in male cohorts (Statement—Table 1e, g; 6d). This is of experimental and practical importance, as variations in adiposity and regional sweat rates [2, 65] between sexes may alter the efficacy and/or safety of menthol containing strategies. This may be especially prudent when applied topically during an exercise bout in an environment where sweat evaporation is the primary avenue for heat loss (e.g., hot/dry, warm/humid conditions). Furthermore, the differences between sexes could be more pronounced following oral application of menthol given identified sex differences in olfaction and trigeminal sensitivity [35]. Inter-individual differences in response to menthol mouth rinsing may also be distinguished by TRPM-8 allele frequency [40], which has shown large regional variation by latitude, but a more practical measure may be the calculation of an individual’s menthol sensitivity index, which describes body-region sensitivity to menthol [47].

The training status of participants is largely homogenous across studies using menthol interventions, typically described as ranging from untrained (i.e., recreationally active) to trained (Statements—Table 1c–f; Statements—Table 6b, c), with limited information presented regarding aerobic fitness (*V̇*O_2max_), training age, or competitive experience. Whilst it is possible that experimental effects are more likely in lesser trained individuals, participants may also demonstrate greater variability in their performances [67]. Hence, extending experimental outcomes from moderately trained to elite athletes, where the efficacy of non-thermal strategies may differ, is inappropriate and requires further experimental and applied research. For example, when considering elite athletes, a recent study reported increases in thermal strain with core temperatures > 39.5 °C in all athletes, with 25% exceeding 40 °C and one above 41 °C [59]. Therefore, the degree of thermal discomfort tolerated/permissible by an elite athlete may far exceed what can be simulated in laboratory-based studies. The motivation to ignore afferent cues of thermal state may be higher in these individuals which may reduce the efficacy of a non-thermal strategy that is thought mainly to alter thermal perception to facilitate ergogenic effects. However, some studies have sought to address the effectiveness of menthol topical application during advanced thermal stress, showing some potency to extend performance [6, 37]. Whilst tests conducted on elite athletes may be desirable due to their improved consistency [67], advantageous performance outcomes may be within the typical error of the test and so may be discredited, although these effects may be of practical importance [11]. Overall, further research is needed to test menthol’s effectiveness in elite populations particularly in the build-up to Tokyo 2021 and beyond.

#### Section Summary and Practical Recommendations

- Menthol research has largely been conducted on male cohorts who are recreationally active or trained.
- Research on female participants is to be encouraged due to potential variations in regional adiposity and sweat rates amongst other factors.
- The use of menthol by elite athletes is currently not well supported; however, its use is unlikely to be detrimental (especially when mouth rinsing unless published protocols regarding frequency and concentration are exceeded)
- Individual effectiveness in representative conditions for forthcoming events should be trialled in the build-up to major competitions to identify athlete specific benefits.

### Experimental Effects

*Perception *Single topical applications of menthol reliably improve thermal perception in a hot environment by lowering thermal sensation [Statement—Table 2b; [4–6, 29, 38, 63] and, in some but not all cases, relieving thermal discomfort [Statement 2a; [4, 6, 29, 63] even at relatively low concentrations (e.g., 0.05% concentration; [4]). Intuitively, the same effects would occur with repeated topical application, although only one completed study to date supports this idea [Statement 2i;[8]. Accordingly, where tolerance to thermal discomfort is a contributing limiting factor for exercise performance, menthol topical application may yield its ergogenic effect through this means. Only studies examining behavioural thermoregulation, rather than explicitly sport performance, have supported this idea [Statements—Table 2d, j; [8, 63]. However, tolerance to increasing thermal discomfort and thus increased ability to maintain exercise intensity may enhance competitive performance [3] particularly at the end of a race. In circumstances where repeated topical application is considered (e.g., training sessions or within races), it should be noted that habituation to repeated menthol topical application (i.e., a diminished response to a stimulus of the same magnitude [24, 30]) has been reported [30]. Therefore, a diminishing return (i.e., lesser perceptual improvement) for each subsequent menthol application is probable [7, 30]. Moreover, the extant skin temperature at the time of menthol application may also be important with skin temperatures above 37 °C conversely suggested to lead to increased sensations of warmth [49].

In athletic events, it is the athlete’s rating of perceived exertion (RPE; [12]) that has been more commonly suggested to limit performance (e.g. [17]). Hence, improvement in RPE (i.e., lower RPE for a given power output) following menthol topical application could confer a competitive advantage. A reduction in RPE was observed during a time-trial following spray application [Statement—Table 2c; [6] and oral application during an RPE-clamp protocol (Statement—Table 7d) saw higher power outputs achieved across the exercise trial [24]. Oral application has demonstrated consistent effects on thermal perception [Statements—Table 7a–c; [24, 28, 37] and RPE [28, 37, 52]. However, differences in the sensitivities of the perceptual scales used to assess thermal perception between studies examining topical and oral application often make direct comparisons of the magnitude of perceptual effects difficult. A word of caution is necessary regarding menthol’s potency in modulating perceptual sensations of exertion or thermal discomfort, whereby the panel reached consensus regarding potentially dangerous side effects. Theoretically maintaining exercise intensity for longer periods in a hot environment could contribute to heat-related illnesses (Statements—Table 7e, g). To our knowledge, there are no recorded instances of heat-related illness in experiments using these interventions; however, practitioners and athletes must consider these associated risks. Laboratory-based thermal physiology experiments abide by strict withdrawal criteria, typically removing participants from hot environments when their core temperature reaches ~ 39.5 °C. Therefore, in less-controlled scenarios, such as during athletic events, careful thermal monitoring of participants is still necessary to avoid heat illness.

*Thermoregulation *The potential for menthol to extend exercise performance in a hot environment is in contrast to an ergolytic effect reported following whole-body topical cream application [44]. These negative side effects were explained by a delayed onset and total gain in sweat production, thereby reducing the capacity to thermoregulate [Statements—Table 2e, f; [16, 29, 44]). During exercise in the heat, maintaining the avenues for heat loss is critical in controlling the risk of heat-related illness. Repeated spray application has also been noted to reduce sweat rate and volume [8]. The resultant side effects of whole-body topical menthol application during exercise in the heat could lead to a greater rate of rise in core body temperature [44] and a higher terminal core body temperature [30], thereby potentiating the risk of hyperthermia. It is important to note that selected topical application in small but sensitive body regions has been shown to elicit 17% improvement in TTE [63]. Therefore, it is important for practitioners and athletes to be aware of key body regions where menthol should be targeted. Nevertheless, at higher core body temperatures, it has been suggested that skin cooling or menthol topical application to the skin may fail to contribute to a reduction in global thermal perception because of an increasingly dominant central nervous system input to thermoreception coming from the hyperthermic core [7, 8].

The effects of menthol topical application on vasomotor responses are less clear, with high concentrations of menthol topical application shown to evoke subcutaneous vasodilatation [16]. Whilst others have reported that vasoconstriction of the subcutaneous vasculature occurs following menthol topical application suggested by lower skin temperatures [5, 29, 31, 44] which we agreed was not associated with menthol mouth rinsing (Statement—Table 7f). Rates of evaporation, dermal absorption, interaction with the medium of menthol delivery (gels vs. liquids), surface area/concentration, and the resultant systemic versus reflex local thermal responses probably account for these different findings. The onward effect on increasing core body temperature was inferred by consensus, irrespective of how this elevation is achieved (i.e., delayed sweating, facilitated evaporation, or vasoconstriction) by agreeing that extended exercise duration in the heat occurs with menthol topical application. Moreover, the resultant effects on thermoregulation may be complicated by topical application of gels or creams which may add insulation to the skin if retained on the skin surface and impair sweat evaporation.

The extent of thermoregulatory responses and alterations in perceived thermal state are, therefore, likely to be related to the concentration of topically applied menthol, the surface area covered, and to be body-region-dependent (Statements—Table 2g, h). Most studies examining topical menthol application have targeted the torso due to a high density of thermoreceptors [33]. Very few studies have explored the relationship between the location on the body stimulated and the evoked change in thermal perception. Lee et al. [47] reported that when a standardised body surface area was stimulated, most sites descriptively favoured differences (cooler sensation) with menthol topical application; here, the chest was reported as being most sensitive. Another study [63] showed that areas such as the neck and face were also reported to be highly sensitive and, in relation to mouth rinsing, the oral cavity is established as one of the most densely innervated parts of the body in terms of peripheral thermal receptors [33]. Hence, oral delivery of menthol maximises its non-thermal cooling properties on perception, but is predominantly menthol concentration-dependent (Statement—Table 7h).

#### Section Summary and Practical Recommendations

- Single and repeated menthol topical applications and mouth rinsing reliably improve thermal sensation and, to a lesser extent, thermal comfort and RPE during exercise in a hot environment.
- Menthol topical application may benefit activities where heat tolerance is limiting, but may increase the risk of heat-related illness.
- Thermoeffector change with menthol topical application is surface area, menthol concentration, and site-dependent, with the chest, face, and neck suggested as the most thermally sensitive areas. No such direct changes to body temperature regulation are evident with mouth rinsing.
- Even at low concentrations, menthol topical application evokes changes in thermoeffector responses, which may increase the risk of heat storage and have deleterious effects on prolonged exercise in the heat.
- Athletes should be familiar with testing protocols and these should be well practiced prior to competitive use. Awareness and education of the potential effects of menthol are critical.

### Health Effects

Despite being a chemical substance, which is accompanied by hazard statements describing potential irritation to skin, serious eye damage, and with the potential for respiratory irritation, menthol is safe at low concentrations (Statement—Table 3a, e; Statement 8b) and is a widely used agent to provide a cooling sensation in a number of everyday and sporting products [10]. Safe menthol doses of 0.1 and 0.5 g L^−1^ have been utilised in mouth rinsing experimental work [66], with topical applications of 0.8% spray [47], and 8.0% gel [63] also tested without adverse effects. Based on animal models (lethal dose = 3300–3400 mg kg^−1^ [Rat/Mouse], 800 mg kg^−1^ [Cat]), a lethal ingestion dose range of 50–150 mg kg^−1^ has been described in humans (Statements—Table 3b, f). This equates to ingestion of menthol crystals or peppermint oil with a mass of 3.5–10.5 g for a 70 kg adult [26]. This range provides a broad estimation, and whilst absolute doses of up to 9 g have been survived, excess ingestion is not without negative implications. Acute ingestion of peppermint oil resulted in coma [55], perhaps via neurological, hepatotoxic [54], or nephrotoxic [42] pathways prior to full recovery following intensive-care treatment. Acute intravenous injection of peppermint oil can cause pulmonary oedema and acute lung injury, due to direct toxicity and a resultant increase in pulmonary vascular permeability [9]. Chronic ingestion of menthol (non-specifically described as consuming “two bags” of menthol rich cough droplets; [10 mg] per droplet) has also been reported. This caused coma and ataxia alongside skin lesions, and gastrointestinal and neurological symptoms in an older individual [1]. Symptoms of this nature have been reported in isolation across experimental models [51, 56], though the most common side effects of menthol are related to allergic contact cheilitis, e.g., dermal reactions to the use of menthol containing lip balms [70]. Further to ingestion-related health issues, which are directly applicable to the end user (e.g., the athlete), individuals preparing menthol should do so adhering to precautionary statement codes (Statements—Table 3c, g), as inhalation (~ 60 min) of peppermint fumes within an enclosed tank can lead to hypoxic brain injury, haematuria, and acute renal failure, secondary to recurrent seizure activity, which ultimately may prove fatal [45].

In spite of these reports describing responses to extreme exposures, this consensus statement affirms that menthol should generally be considered as a substance that presents little harm to the user (Statements—Table 3a, e), but is probably not yet widely used in sporting activities (Statements—Tables 3d, h; 8a, d). Adhering to safe working practices, such as but not limited to: preparing mouth rinses from crystals in ventilated environments, applying modest (but not excessive) topical applications in accordance with manufacturer instructions, and delimiting doses to those that have been examined in the peer-reviewed literature should ensure menthol topical application or mouth rinsing minimises the risk to the end user. Given the more likely negative health effect from ingestion (vs. topical application), guidelines proposing a mouth rinse or a beverage containing menthol (0.1–0.5 g of crushed menthol crystals in 1 L of water) should be followed [Statement—Table 8c; [66].

As with any intervention, the use of menthol should be trialled extensively in training to determine both the ergogenic efficacy and safe implementation using verified, food-grade products [66]. It is noteworthy within rinsing protocols that a clear dose–response has yet to be identified, and thus, individual approaches are warranted above pursuing a ‘more is better’ approach [11]. As with thermal cooling strategies, the influence of perceptual cooling on pacing and subsequent thermoregulatory responses, i.e., increased self-selected work rate, and concurrent increases in heat production should also be examined [27]. Perceptual cooling via menthol should not be seen as an alternative to thermal cooling based on the potential to induce reductions in perceived temperature. Menthol does not appear to offer any enhanced heat loss properties, despite acting upon smooth muscle, and thus, in scenarios where reducing the physiological temperature of an individual is required, well-rehearsed recommended strategies for performance enhancement [62, 68] and treatment of heat illness [15, 46] should be implemented.

#### Section Summary and Practical Recommendations

- Case reports exist highlighting potentially detrimental health effects.
- When used in a manner replicating peer-reviewed experimental work, topical and ingested (mouth rinsing) menthol is safe.
- For both single and repeated topical applications, topical menthol should be applied as directed in manufacturer guidelines.
- For both single and repeated uses, menthol mouth rinses should be prepared using food-grade substances provided with certification of purity in well-ventilated spaces.

### Spirit of the Sport

Any nutritional or pharmacological substance with the potential to enhance athletic performance immediately raise questions relating to the ethics of its use during competition and, thus, may be subject to consideration in the context of anti-doping [57]. An abridged version of the World Anti-Doping Agency (WADA) criteria for ensuring that the ‘Spirit of Sport’ is not contravened states that a substance must not fulfil two of three criteria; (1) has the potential to enhance/enhances sport performance, (2) use represents an actual or potential health risk to the athlete, (3) violates the spirit of sport described in the introduction to the Code. Based on published evidence, we agreed that criteria and (1) and (2) could be violated by the use of menthol (Statements—Table 4a, b, e, f; 9a, b); however, we do not yet agree that menthol in all its forms should be banned as the evidence base for its use is still evolving (Statements—Table 4d, h; 9c). Furthermore, in 2004 caffeine, a substance previously banned above urinary concentrations > 12 µg mL^−1^ was withdrawn from WADA’s banned substance list, in part due to common presence/applications in beverages, food stuffs, and some over-the-counter medicines; menthol shares some of these common presences/applications. Consequently, the idea that topical application and mouth rinsing menthol modalities violate the spirit of sport or provides an unfair advantage did not attain consensus (Statements—Table 4d, g; 9h); menthol is widely used in everyday items such as toothpaste and foodstuffs. This likely reflects some inconsistency in reporting of ergogenic effects and acknowledges the commercial and natural availability of menthol. At the present time, authors within this consensus statement acknowledge that both single and/or repeated use of topical or mouth rinse forms of menthol appear not to be widespread (Statements—Tables 3d, h; 8a, d) and, thus, consideration for scrutiny by WADA is likely to be contingent on increasing applied interest in the substance.

#### Section Summary and Practical Recommendations

- Menthol is widely accessible and commercially available in various forms, and thus, it does not permit unfair sporting advantage to some potential users.
- Menthol topical application and mouth rinsing probably do not confer an unfair advantage.
- Menthol topical application and mouth rinsing do not violate the spirit of the sport.
- When using menthol, athletes should avoid combining menthol with other nutritional or pharmacological substances, which may render it ineffective, harmful, or give the potential for menthol to be contaminated with unapproved substances.

### Levels of Evidence

*Quality of research design *Randomised, controlled trials (RCT) and meta-analyses (which integrate data from a range of studies) are considered the highest level of scientific evidence. In the menthol literature, a meta-analysis published in 2019 [38] and a systematic review published in 2017 [66], largely agreed that internal application of menthol (e.g., mouth rinsing) was a more consistently effective strategy than external applications to facilitate athletic performance. Experimental research into menthol across the field generally utilise the repeated-measures design protocol (Statements—Tables 5c, e; 9h) where the same participants take part in each condition of the experiment, thus controlling for participant variability. However, poor reporting of randomisation procedures for each condition examined may increase the experimental bias across the field. It is also evident that repeated topical application of menthol relative to an acute topical application is also largely underreported, with no clear consensus on whether repeated topical application *sustains* an ergogenic effect. Therefore, greater diversity in research design, experimental questions, and clear reporting of randomisation procedures is needed. In addition, most research is laboratory-based (Statements—Tables 5a, d; 9e, f), with limited examples of in-field topical application of menthol in a sporting context. Thus, the question of ecological validity when applying largely laboratory-based outcomes to real-world sporting events requires future work in an applied context.

*Experimental bias *Experimental bias can occur when experimenters expect to find a particular result. It is, therefore, good practice to use blinding procedures. Working double-blind ensures that both the participant and investigator are unaware of each condition; however, limited studies have adopted this procedure, with only one reporting double blinding [6]. The majority of studies adopt a single-blind approach, where the participant is unaware but not the investigator, which is associated with a risk of increased bias. A number of studies have not adequately reported blinding procedures [e.g., 4, 44, 69] and, therefore, future studies should attempt to fully disclose the procedures used in the experimental design to enable consistency in the field. However, it should be noted in relation to blinding when implementing menthol mouth rinsing, that a key concern is adequately controlling for its distinctive sensory effect; it has proved more straight forward in menthol topical application studies [e.g., 4, 5, 6, 8, 29, 30] to include a comparable control (Statement—Table 5b). Whilst suitable placebos are not available, some researchers examining mouth-rinsing have used a strong orange-flavoured [52] or bitter apple-flavoured [24] placebo solution to stimulate the oral cavity which was considered appropriate by the panel (Statement—Table 9g). Irrespective, it must be made clear when designing future studies that the participant must remain naive to the original research question to appropriately assess menthol’s efficacy in an unbiased manner.

#### Section Summary and Practical Recommendations

To make research findings applicable to athletes in competition:

- Ensure adequate experimental design and blinding.
- Include a representative control condition.
- A true placebo is required.

### Future Research Directions

Whilst there is sufficient evidence to support the use of menthol to enhance endurance exercise performance in controlled hot environments, the current consensus has identified a number of areas for future research. Perhaps of highest priority is the need for further research among elite athletes, particularly in events that are representative of ‘real-world’ performance. Whilst it is feasible that the perceptual responses of elite athletes to menthol topical application or rinsing are similar to that of sub-elite populations, it is not known whether these subtle alterations will translate to measurable outcomes in the field. Indeed, it is not yet apparent whether the small-to-moderate effects elicited in laboratory-based capacity tests relate to field-based time-trial performance improvements, which accounts for the majority of Olympic Sport and relates to other endurance events. Moreover, the effects on speed and strength are also worthy of future consideration. Irrespective of the focal pursuit of the onward research, there is an under-representation of female participants in the current literature, which must be rectified in future owing to potential differences in the sensitivity of response to various stimuli in the oral cavity and variation in thermoregulatory behaviour between sexes.

The acute physiological effects elicited by menthol are dependent upon the mode of administration, with topical application potentially leading to altered thermoeffector response and potential heat gain. Given the reliance upon evaporative and convective cooling avenues during endurance exercise in the heat, coupled with the likelihood of high heat indices during the Tokyo 2021 Olympic games and similar events, these effects would be undesirable and may have deleterious performance or health outcomes. Therefore, further work is required to establish the optimal type (e.g., dose, timing, concentration, and target body surface area) of menthol administration in field-based scenarios, particularly when balanced against the logistical limitations that are imposed during a given event, which may limit the scope for repeated use. If mouth rinsing is the preferred form of menthol administration for athletes in the field, there is a need to understand the reasons why repeated mouth rinsing might result in diminishing performance returns and whether manipulations of menthol dose and timing help in sustaining its cooling effects. This would further the current understanding of optimal supplementation strategies among athletes and will help to inform elements of race-day planning. Finally, exploring the additive effects of other TRPM-8 agonists that are not competitive with menthol [64] may produce synergistic experimental effects to those of menthol alone.

#### Section Summary and Practical Recommendations

- Studies are required using menthol with elite athletes during exercise in the heat.
- Ecologically valid field-based research is required to support the abundant laboratory-based evidence.
- Further research is required among females of all training backgrounds.
- Further understanding of single or repeated mouth rinsing and the reasons for diminishing returns need to be clarified.

## Conclusion

Menthol topical application and mouth rinsing are ergogenic in hot environments, improving performance and perception, with differing effects on body temperature regulation. Evidence shows that these interventions improve endurance performance, but that further work is required to establish their effects in other sports and activities specific to the Olympic games. There is a particular shortage of evidence on their effects in females and in elite athletes. When applied, rinsed, or ingested at a high concentration and volume, menthol can be harmful to health. We provide important practical advice based on an expert led consensus process for the safe and effective use of menthol for athletes, practitioners, and researchers. Consequently, athletes and federations can begin to safely explore the possible benefits to elite sport performance for the Tokyo 2021 Olympics, which will take place in hot (~ 31 °C), humid (70% RH) conditions.

## Data Availability

The dataset generated during and analysed during the current study are available in the Leeds Trinity University, PURE Institutional repository, https://research.leedstrinity.ac.uk/en/publications/menthol-as-an-ergogenic-aid-for-the-tokyo-2021-olympic-games-an-expert-led-consensus-statement-using-the-modified-delphi-method(7a4e8d11-b997-4cc6-b2ef-b6e6927997b8).html.
